# Robotic monitoring of grasslands: a dataset from the EU Natura2000 habitat 6210* in the central Apennines (Italy)

**DOI:** 10.1038/s41597-023-02312-x

**Published:** 2023-06-27

**Authors:** Franco Angelini, Mathew J. Pollayil, Federica Bonini, Daniela Gigante, Manolo Garabini

**Affiliations:** 1 Centro di Ricerca “Enrico Piaggio”, and Dipartimento di Ingegneria dell’Informazione, Università di Pisa, Largo Lucio Lazzarino 1, 56122 Pisa, Italy; 2 Department of Agricultural, Food and Environmental Sciences, University of Perugia, Borgo XX giugno 74, I-06121 Perugia, Italy

**Keywords:** Electrical and electronic engineering, Grassland ecology, Biodiversity

## Abstract

Despite the remarkable growth of the global market for robotics, robotic monitoring of habitats is still an understudied topic. This is true, among others, for the species-rich EU Annex I habitat “6210 - Semi-natural grasslands and scrubland facies on calcareous substrates”. This habitat is typically surveyed by human operators. In this work, we present a dataset concerning relevés performed through the quadrupedal robot ANYmal C. The dataset contains information from three plots, which include the robot state, videos, and images acquired to assess the habitat conservation status. Additionally, a collection of videos and pictures about two typical and one early warning species of habitat 6210 is also presented. This database is publicly available in the provided Zenodo repository and will aid researchers in several fields. Robot state information can be used by engineers to validate their algorithms, while data gathered by the robot can be used to design new methodologies and new metrics to assess the habitat conservation status or train/test classifiers (e.g. neural networks) for plant classification.

## Background & Summary

Environmental degradation and climate change represent a significant threat to life. To address these, the European Union (EU) established the Green Deal^1^, a set of policies aimed at making the EU the first climate-neutral continent by 2050. The protection of biodiversity is one of the topics covered by these policies, and the EU has strived towards conservation initiatives to stop the loss of biodiversity. Specifically, based on the European Directive 92/43/EC “Habitat”^2^, EU created the Natura 2000 Network, one of the world’s largest systems of protected lands^3–5^. The evaluation of the conservation status of the habitats and species included in the Annexes to the “Habitat” Directive is crucial for preventing biodiversity loss^6–8^.

According to the “Habitat” Directive, the conservation status of each habitat type has to be assessed by four criteria (area, range, structure and functions, and future prospects). Among them, the “structure and functions” parameters refer to the physiognomical/structural component of the habitat and the ecological processes occurring at different temporal and spatial scales which are necessary for the long-term maintenance of habitat itself^6^. The European and national guidelines^6,9^ state that these parameters can be monitored combining floristic and vegetation data collected at local scale (e.g. Natura 2000 sites).

In this work we focus on Annex I habitat “6210(*) Semi-natural dry grasslands and scrubland facies on calcareous substrates (*Festuco-Brometalia*) (*important orchid sites)”, one of the most diverse and species-rich in the world, after tropical rainforests^10^, whose maintenance depends on traditional practices such as extensive grazing and mowing^11^. Monitoring the “structure and functions” of this habitat entails the recording of several parameters, such as total vegetation cover, presence and coverage of dominant species, typical species (in particular orchid species), indicator species of disturbance and dynamic phenomena, alien species^8,12^. Typical species, hereinafter TS, “should be selected to reflect favourable structure and functions of the habitat type”^6^. This means that, TS should simultaneously be: i) a good indicator of the favourable habitat quality; ii) unique of the habitat or present over a broad portion of the habitat range; iii) sensitive to changes in the habitat conditions^6,13^. Actually, given the variability of Annex I habitats, and in particular the wide floristic diversity of grasslands, there are no officially recommended lists of TS, neither at European nor at national scale, and it is recommended to point out the target TS at regional or even local scale^14^. The presence of orchid species is generally considered as an indicator of favorable conservation status, as well as their eventual abundance in the recorded area is particularly relevant as indicator of the priority status of the habitat 6210^15–19^.

The concept of “early warning species” (hereinafter EWS) applies to those plants whose settlement in the habitat patches is an indication of ongoing processes altering the structure and function of the habitat itself; this concept applies also (perhaps mostly) to non-typical taxa, whose settlement can be a clear sign of transformation. In this sense, non-typical EWS can be used as very informative proxies, pointing out the first community functional shifts^20^. Among the signals addressing grassland habitat degradation, the appearance and establishment of edge and mantle species is considered an important early warning indicator that should be monitored, as it represents ongoing dynamic processes that can lead, in a more or less short time, to a complete habitat alteration^9^.

The standard collection of vegetation data suitable for the assessment of grassland conservation status is generally performed by a sampling field relevé^9,14^. According to the national standards^9,14^, the relevé must be carried out in randomly located 4 × 4 m^2^ homogeneous plots, whose number is proportional to the total habitat surface. The recommended area 4 × 4 m^2^ complies with the EU standards for grasslands sampling^21^. The data acquisition and assessment of the habitat conservation status are typically performed only by human operators, a time and cost inefficient approach. Additionally, funding for biodiversity preservation is often not adequate^22,23^. Given these observations, the goal of the Natural Intelligence project (https://www.nih2020.eu/) is to enrich the human monitoring capabilities through the use of robotic systems. In particular, we use legged robots to acquire information on the habitat mimicking the surveys performed by humans. The choice of this type of robot is a trade-off between mobility and battery duration.

**Fig. 1:**
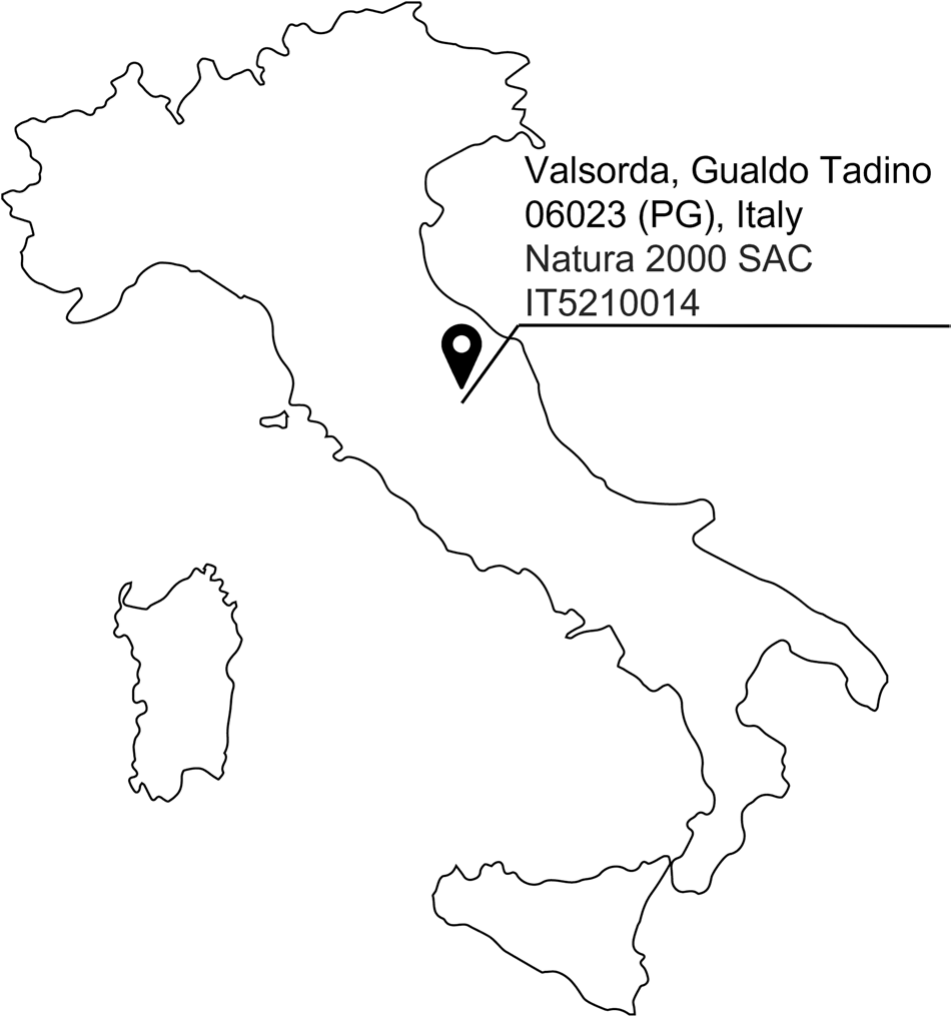
Location of the data acquisition campaign.

**Fig. 2:**
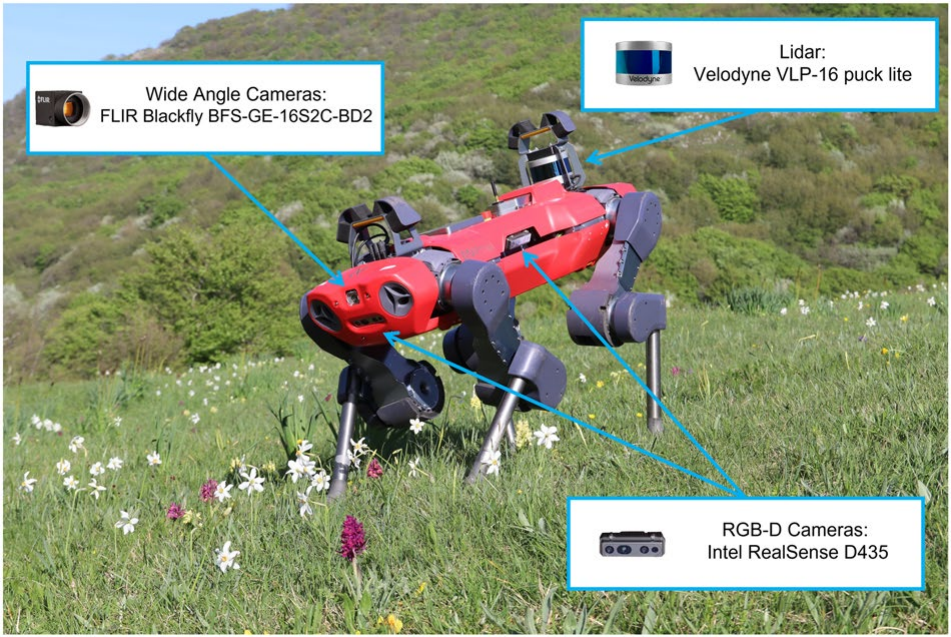
ANYmal C robot collecting data in the habitat 6210. The picture highlights the sensors equipped on the robot for data collection: one Velodyne VLP-16 puck lite, two FLIR Blackfly BFS-GE-16S2C-BD2 wide angle cameras, and four Intel RealSense D435 RGB-D cameras.

In this dataset, we report the data collected during a monitoring mission in the occurrences of the habitat 6210 in Valsorda, Gualdo Tadino (PG), Italy (Fig. 1), inside the Natura 2000 SAC IT5210014, employing a quadrupedal robot ANYmal C^24^ (Fig. 2). The data have been collected by a team of robotic engineers and plant scientists. The information included in this dataset can be divided into two groups. The first one is a collection of videos and images of three indicator plant species acquired from the robot cameras. The second group includes data related to the autonomous monitoring missions. Specifically, we report the point cloud of the area, the robot state information, and the acquired videos and pictures.

This dataset has a multidisciplinary scope and can be used by researchers in several fields. For instance, point clouds and information about the robot state could be used by robotic engineers to test or validate their own methods as well as benchmark the robot performance. On the other hand, plant videos and images recorded by the robot could be used by botanists to assess the quality of this information as well as the habitat’s condition, or by computer scientists interested in testing their Artificial Intelligence (AI) algorithms for species detection and classification, as well as for exploring new possibilities for integrated human-AI monitoring.

To the best of authors’ knowledge, this is the first publicly available dataset on robotic habitat monitoring.

## Methods

The gathering of the full dataset - comprising monitoring missions and typical and early warning indicator species - was carried out by a team composed of both robotic engineers and plant scientists in Valsorda, Gualdo Tadino 06023 (PG), Italy, inside the Natura 2000 SAC IT5210014 (Fig. 1). This location was chosen because it represents a notable stand of the target habitat 6210 in central Apennines. The period of data acquisition was from 10th to 13th of May, 2022. This month was selected because, as stated in the national guidelines for the habitat monitoring^12^, the optimal sampling period for habitat 6210 covers the time range between May and August, when the majority of the composing species reach their maximum development and perform flowering. Considering the particular role played by orchid species in this habitat type^15^ and based on knowledge of local flora and vegetation, the data collection has been executed in May as an ideal period to detect both the target TS and EWS species in the field. May is the best period to monitor their occurrence, since they all typically flower between April and June^25^. In particular, orchid species are practically indistinguishable in the early stages of their life cycle, when only basal leaves occur, but become clearly recognizable at the time of flowering.

The platform used for data acquisition is the quadrupedal robot ANYmal C^24^ (Fig. 2) produced by ANYbotics AG. This robot can move both in an autonomous or teleoperated manner. Information about the environment are collected through a LiDAR sensor and four RGB-D cameras. The former is a LiDAR Velodyne VLP-16 puck lite (https://velodynelidar.com/products/puck-lite/), which acquires a 3D map of the environment. The cameras, instead, are four Intel RealSense D435 cameras (https://www.intelrealsense.com/depth-camera-d435/) that are able to collect full HD RGB images and/or 30 fps videos. Figure 2 depicts the location of the sensors: the LIDAR is placed on the rear part of the system, while the four cameras are placed one per side. The platform is equipped also with two wide angle FLIR Blackfly BFS-GE-16S2C-BD2 cameras, which were not used for data gathering. The information about the robot state are collected via the ROS interface of the robot and saved as ROS bag files. For more details about this particular file type please see the official ROS bag reference (http://wiki.ros.org/rosbag).

The dataset contains two different sets of data: (i) typical and early warning species data and (ii) monitoring mission data. Both sets of data are collected with the same device and are related to habitat 6210. However, their content and goal are different. The first batch of data contains categorized pictures and videos of indicator species for habitat 6210. Each species occurrence has been chosen independently and has the only goal of being an example of the indicator species. The second batch of data contains information about habitat surveys performed by the robot using procedures similar to the ones followed by botanists. Both methodologies are described in detail in the following sections.

### Typical and early warning species data

This part of the dataset includes pictures and videos of indicator species for habitat 6210. We selected as indicator species three plant taxa with a key role as explanatory proxies of the habitat 6210 conservation status. They are specifically two TS and one non-typical EWS. All data were categorized by botanists expert of the local flora.

Following the guidelines in^6^, and in accordance with the suggestion to select the TS of the habitat 6210 at local level^12,14^, we selected two typical orchid species, among those mentioned in the national Interpretation Manual^16^: *Anacamptis morio* (L.) R.M.Bateman, Pridgeon & M.W.Chase and *Dactylorhiza sambucina* (L.) Soó (with both the yellow and pink forms). Furthermore the selected TS are among the most frequent species of orchids occurring in the habitat 6210 at regional level^26^.

For what concerns EWS, in the Apenninic extents of habitat 6210, emblematic examples are *Asphodelus macrocarpus* Parl., *Pteridium aquilinum* (L.) Kuhn, *Brachypodium rupestre* (Host) Roem. & Schult., and *Brachypodium genuense* (DC.) Roem. & Schult^27–29^. Among these species, we chose *Asphodelus macrocarpus* Parl., a rhizomatous geophytes species with rapid vegetative growth, which typically expands from heliophilous forest edges colonizing grassland habitats. The occurrence and the impact of this species on habitat 6210 in the central Apennines is well documented, especially when traditional agropastoral activities are underpracticed or abandoned^27,30,31^. Please note that the nomenclature of plant species is in accordance with the World Flora Online portal^32^.

**Fig. 3:**
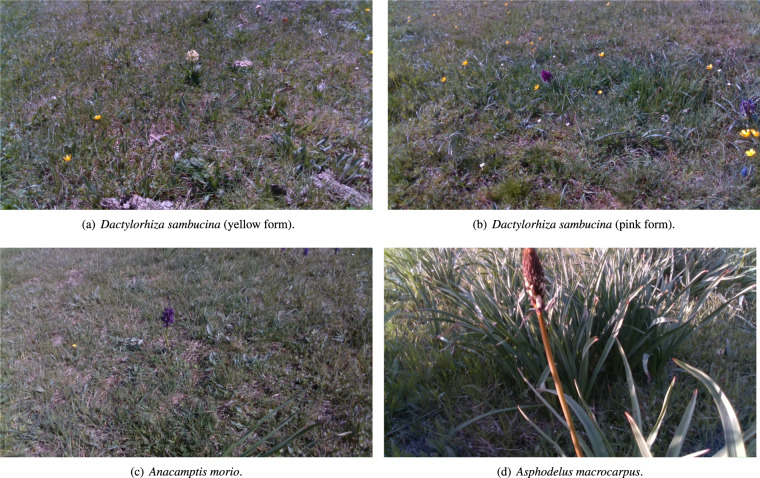
Examples of species pictures taken by the robot. (**a**–**c**) are typical species, while (**d**) is an early warning species.

To acquire the data, first, plant scientists identified the instances of the indicator species in the study area employing the diagnostic keys in^25^. Then, an expert human teleoperated the robot, placing it in front of the chosen instance. This may include one or more of the three species. Finally, the video acquisition was manually started and a video of at least 900 frames was recorded together with 10 pictures. This procedure has been performed 16 times for each of the indicator species. In the case of *Dactylorhiza sambucina*, we executed the procedure 16 times for each form: pink and yellow. Pictures and videos contain at least one instance of the indicator species, but they may contain also instances of other species. Table 1 summarizes the entries of this part of the dataset, while Fig. 3 shows a picture example for each of the indicator species.

**Table 1:** Name of the selected four typical species of the habitat 6210 and number of videos and pictures for each species.

Name	Type	# videos	# pictures
*Dactylorhiza sambucina* (L.) Soó (pink form)	Typical species	16	151
*Dactylorhiza sambucina* (L.) Soó (yellow form)	Typical species	16	161
*Anacamptis morio* (L.) R.M.Bateman, Pridgeon & M.W.Chase	Typical species	16	161
*Asphodelus macrocarpus* Parl.	Early warning species	16	161

The peculiarity of this part of the dataset is that, to the best of Author’s knowledge, this is the first dataset of classified images and videos of indicator species for habitat 6210 taken by a quadrupedal robot. The main goal of this batch of data is to publicly share data that can be used to design novel classification algorithms or to test on robot-acquired data already developed methods. Given this objective, no other information is provided about these data.

### Monitoring mission data

This other part of the dataset contains information about the monitoring missions. The idea is to use the quadrupedal robot ANYmal C to imitate the survey of a standard plot by botanists during field relevé. This means that the robot follows the same standard procedures and collects information about the area, but it does not analyze nor classify it.

Each area to be surveyed is randomly chosen, and the GPS coordinates are recorded by a human operator employing a Garmin GPS Etrex 10. Each coordinate has been recorded with an accuracy of at least 3 m. In addition to location information, also the time, the date and the weather conditions have been noted. Georeferencing is utterly important to compare data with past or future surveys, while date and weather information are useful for having an approximate estimate of the sunlight. Time and date were automatically saved with the robot status, while weather was visually inspected and manually noted.

The actual survey is divided into two phases: (i) mapping, and (ii) autonomous monitoring mission. We define as mapping the creation of a 3D map of the environment. Indeed, in this phase, the LiDAR sensor mounted on the robot is used to scan the surrounding area. During this scanning, the laser sensor determines the distance between the robot position and the obstacles. To improve the digital representation of the environment, an expert human operator guides the robot around, while the LiDAR gathers information. The result of this procedure is a set of points, namely point cloud, which represents the environment, in terms of both terrain and potential obstacles. An external video was also recorded to qualitatively show the whole phase.

**Fig. 4:**
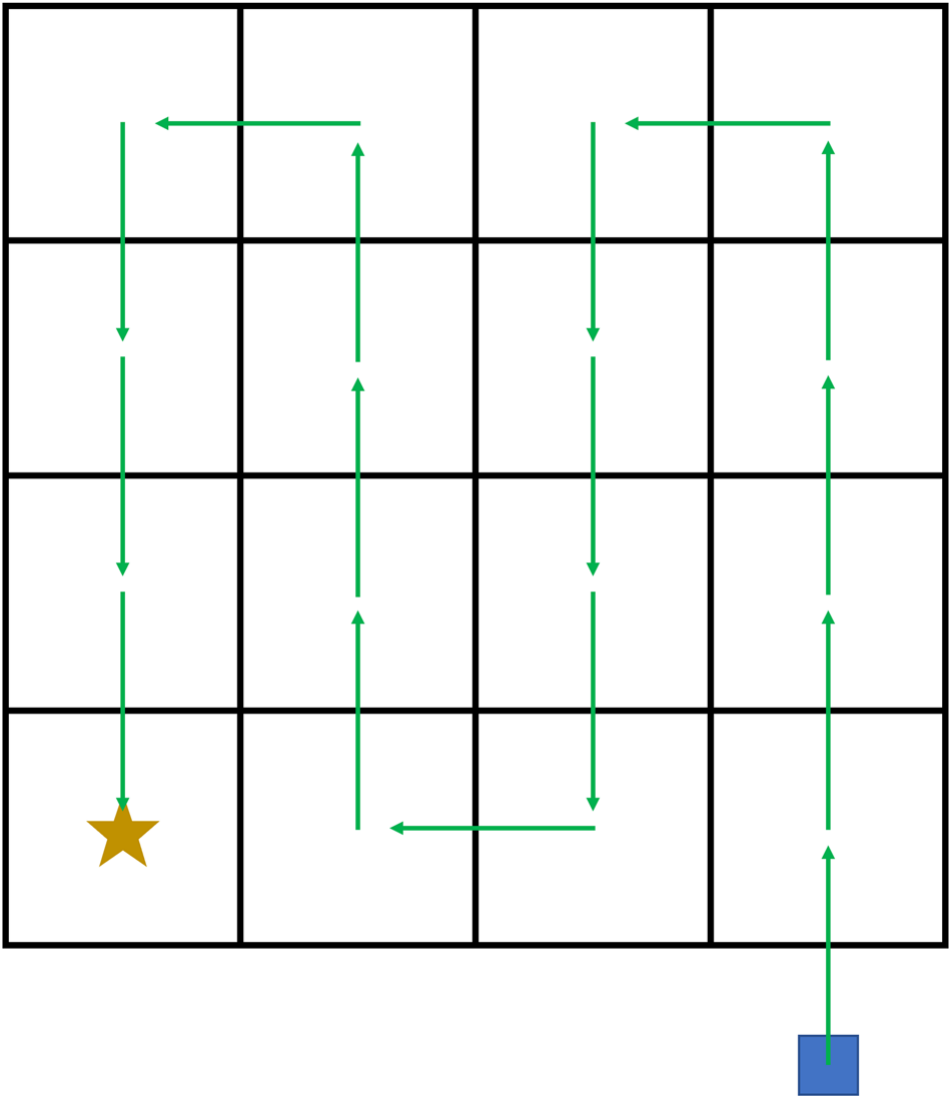
Motion of the robot during the autonomous monitoring mission. The robot starts on the blue square out of the grid. Green arrows indicate the motion of the robot. At each waypoint, i.e., the center of each square, the robot stops and take pictures with all cameras. The yellow star is the final position of the monitoring mission. During the whole mission duration the cameras recorded a video, and the robot status information have been saved.

The point cloud obtained in phase (i) is essential to enable the robot self localization, which is fundamental to enable the autonomous locomotion. The latter is crucial for phase (ii), i.e., autonomous monitoring missions. In this phase, the robot mimics the habitat relevé typically executed by plant scientists. This means that the robot acquires information on plots with the same size of those surveyed by botanists following the national and international standards. In accordance with these standards^9,21^, the area to be monitored is 4 × 4 m^2^, and ANYmal completely examines it. To do that, the robot autonomously moves on a grid, following a set of waypoints that starts on the bottom right corner of the area and ends on the top right one. Figure 4 schematizes the robot motion. During this phase the robot keeps its orientation constant. When a waypoint is reached, i.e., in the center of each square in Fig. 4, the robot stops and takes a picture from each of the four RGB-D cameras. Each camera records also a video for the whole mission duration. In addition to these data, in this phase we recorded also the robot status information and an external video of the whole robot motion. No analysis is performed on the robot-acquired data.

Table 2 reports information about the three monitoring missions we performed. Since the three areas were close, a single mapping phase was sufficient to enable all autonomous monitoring missions. Table 3 summarizes the robot status information. These are collected using ROS bag files of several topics of the robot control architecture. Robot status information includes, for instance, robot base position, joint position, joint velocities, joint acceleration, joint torque, joint current, and battery status. Details about the topics and the specifications on the datastream output from the ANYmal C robot are available on the website of ANYmal Research (https://www.anymal-research.org/). Figure 6 is an example of (part of) the robot status during part of Plot 1.

**Table 2:** Date, time, weather information, and georeferencing information of the autonomous monitoring missions.

Name	Date	Time	Weather	Latitude	Longitude
Mapping	11 May 2022	—	Sunny	—	—
Plot1	11 May 2022	17:48	Sunny	43°15′34.39″N	12°48′57.94″E
Plot2	11 May 2022	18:00	Sunny	43°15′33.97″N	12°48′57.81″E
Plot3	12 May 2022	10:52	Sunny	43°15′34.70″N	12°48′57.70″E

**Table 3:** ROS topics recorded during robot motion.

Topic Name	Description
/state_estimator/anymal_state	Robot info, e.g., base position [m] and orientation [rad], joint position [rad], joint velocity [rad/s], joint acceleration [rad/s^2^], joint torque [Nm], etc.
/log/state/current/LF_HAA	Current [A] of the hip adduction/abduction joint of the left fore leg
/log/state/current/LF_HFE	Current [A] of the hip flexion/extension joint of the left fore leg
/log/state/current/LF_KFE	Current [A] of the knee flexion/extension joint of the left fore leg
/log/state/current/LH_HAA	Current [A] of the hip adduction/abduction joint of the left hind leg
/log/state/current/LH_HFE	Current [A] of the hip flexion/extension joint of the left hind leg
/log/state/current/LH_KFE	Current [A] of the knee flexion/extension joint of the left hind leg
/log/state/current/RF_HAA	Current [A] of the hip adduction/abduction joint of the right fore leg
/log/state/current/RF_HFE	Current [A] of the hip flexion/extension joint of the right fore leg
/log/state/current/RF_KFE	Current [A] of the knee flexion/extension joint of the right fore leg
/log/state/current/RH_HAA	Current [A] of the hip adduction/abduction joint of the right hind leg
/log/state/current/RH_HFE	Current [A] of the hip flexion/extension joint of the right hind leg
/log/state/current/RH_KFE	Current [A] of the knee flexion/extension joint of the right hind leg
/pdb/battery_state_ros	Battery information, e.g., charge percentage, voltage [V], current [A], etc.
/path_planning_and_following/navigate_to_goal/result	Info on the success (or failure) of the robot in reaching the desired navigation goal
/path_planning_and_following/trajectory_poses	The planned Cartesian poses (waypoints) tracked by the robot to reach the goal
/path_planning_and_following/active_path	The actual Cartesian path through the waypoints followed by the robot to reach the goal
/tf	Coordinate frames and transformations between them (TFs) (http://wiki.ros.org/tf2)

## Data Records

In this section we describe the data contained in this dataset, we list the different file formats, and we explain how to visualize them. All data are uploaded on Zenodo^33^ at 10.5281/zenodo.7385369, while an example code to extract the bag files is uploaded on Zenodo^34^ and GitHub^35^.

**Fig. 5:**
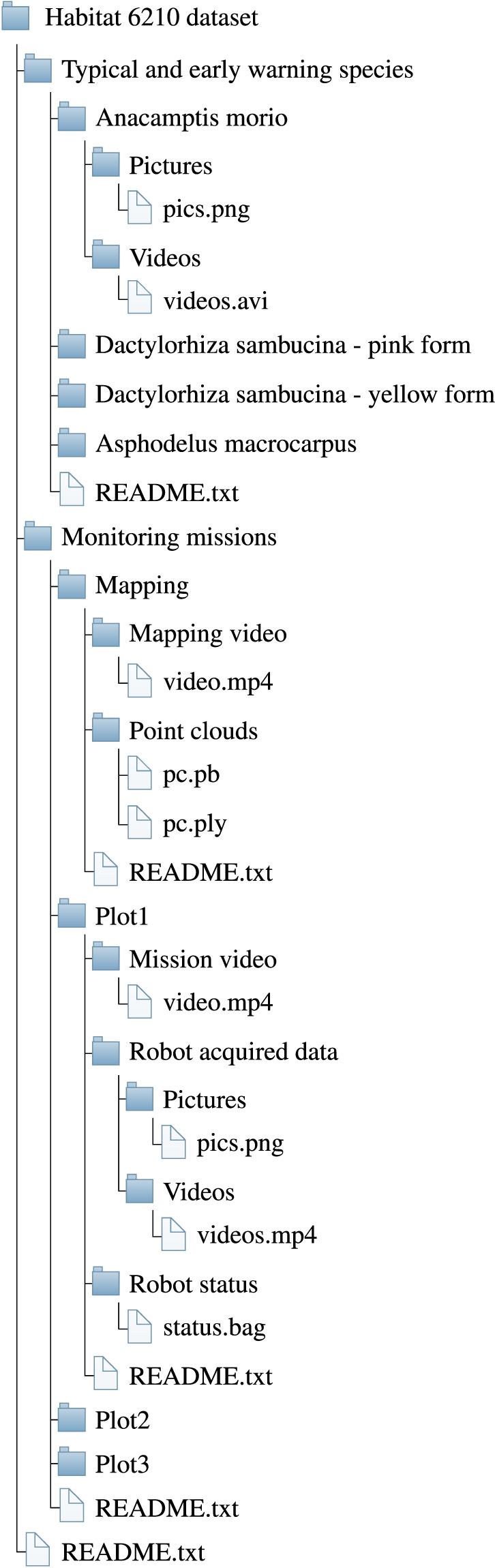
Directory structure tree of the dataset.

The tree structure of the full dataset is depicted in Fig. 5. We provide two sets of data together to a README.txt file to navigate through them. The first dataset, named “Typical and early warning species”, is a collection of videos and images of three indicator species, which are TS or EWS for habitat 6210. A README.txt file explains the structure of this set of data. For each species, we provide a “Pictures” and a “Video” folder containing the pictures and the videos of the indicator species. Each species has been recorded 16 times. Table 1 summarizes the number of entries of this set of data.

**Fig. 6:**
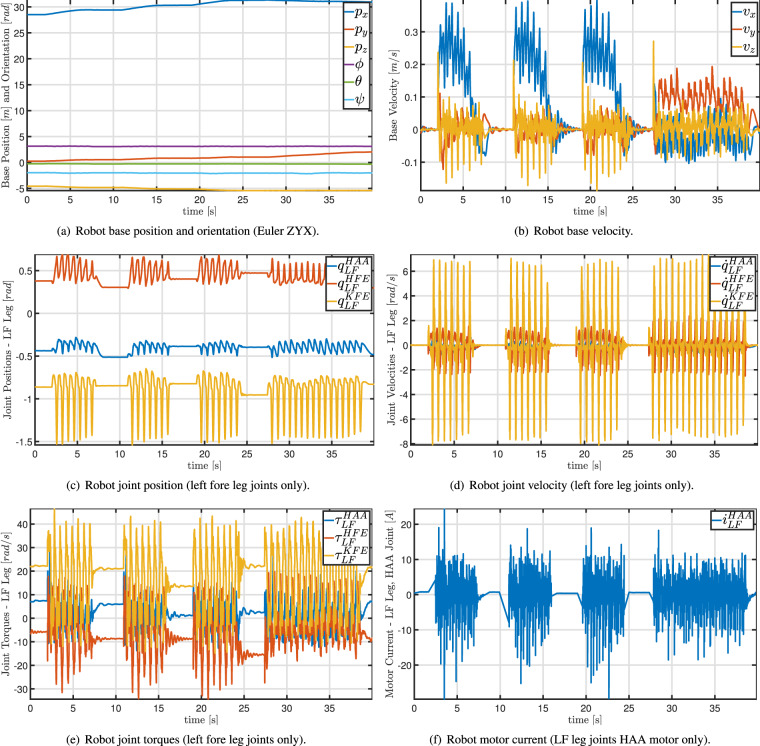
Examples of graphs regarding data extracted from the ROS bag file of Plot 1 (see Fig. 5) performed as in Fig. 4. See also the provided MATLAB code. For the sake of image clarity, only approximately a quarter portion of the entire mission (40 s out 140 s) is shown in these graphs.

The second one, named “Monitoring missions”, contains data related to the mapping and the three monitoring missions. A README.txt file explains the structure of this set of data. The “Mapping” folder contains a README.txt file, a video of the mapping operation and the acquired point cloud. Each of the three “Plot” folders contains three subfolders and a README.txt file with time and date at the start, together with weather information. The three subfolders contain a qualitative video of the operation, the ROS bag files related to the robot state, and the videos and images acquired by the robot. The latter data are named based on the camera used to acquire the information. Specifically, “depth_front”, “depth_left”, “depth_rear”, and “depth_right” refer to the RGB-D camera placed on the front, left, rear, and right side of the robot, respectively. The bag files are instead named with the date and time at the start of the recording.

### Data formats

In the following we distinguish data elements depending on the file format.

#### .jpg

This is a standard file format for images and can be visioned using many classic image viewer software. The jpg files in the dataset are only the images collected by the robot, both during autonomous monitoring missions and during typical and early warning species data gathering.

#### .mp4 and .avi

These are standard file format for videos and can be visioned using many classic multimedia software. The avi files refer to videos collected by the robot, both during autonomous monitoring missions and during typical and early warning species data gathering. The mp4 files are the videos taken with a reflex digital camera and their goal is only to qualitatively see the robot motion.

#### .ply and .pb

Point clouds have been saved in two formats: ply and pb. Polygon File Format (ply) is a standard format for 3D models, and it can be opened with several software s, e.g., MATLAB (https://www.mathworks.com/products/matlab.html), but there are also free alternatives like MeshLab. pb format is the default one to save point clouds with ROS Gazebo ANYmal research (https://www.anymal-research.org/), and also the one used to load pointclouds. Thus, depending on the goal, users can prefer ply or pb file format.

#### .bag

This is the standard format that ROS employs to store robot data. The dataset contains bag files related to autonomous monitoring missions. This data can be visioned through several ROS packages, but also through other means, for instance, using data processing software like MATLAB (https://www.mathworks.com/products/matlab.html). In this latter case, a typical procedure is the following. The bag files contain data saved from the so-called ROS topics (http://wiki.ros.org/rostopic), which are streams of data in the form of ROS messages. The particular list of topics that we saved is shown and described in Table 3. These topics contain all the relevant information about the state of the robot and its main components.

### Example code for data analysis

The robot status is stored as bag files. The extraction of the data from bag files can be performed using many ROS packages, or other softwares, e.g., MATLAB (https://www.mathworks.com/products/matlab.html). In Code 1, we provide a minimal example of MATLAB script to analyse the data contained in bag files. To run this example, it is required to have MATLAB (https://www.mathworks.com/products/matlab.html) and ROS toolbox (https://www.mathworks.com/products/ros.html). This code was written and tested on MATLAB R2022a, but it may also work on later MATLAB releases. The function extracts data as a MATLAB structure from a ROS bag file from a specified topic. Again, the list of topics that are available in the data we provide is shown in Table 3. More detailed descriptions of the topics and the specifications on the datastream output from the ANYmal C robot are available on the website of ANYmal Research (https://www.anymal-research.org/), which however can only be accessed by research institutions after a partnership request is accepted by ANYbotics. This notwithstanding, please note that the provided data can be freely accessed without the need for registering to ANYmal Research. Additionally, the code provided in the following section is sufficient to visualize and analyze the data. Partnership with ANYmal Research is only to receive very specific details about the robot and is not required to access and analyze the provided data.

#### Code 1

Example MATLAB code to extract data from ROS bags.

## Technical Validation

Quality assurance during the fieldwork was provided both by the robotic engineers team, i.e., Franco Angelini (FA), Mathew Jose Pollayil (MJP), and Manolo Garabini (MG), and by the plant scientists team, i.e., Federica Bonini (FB), and Daniela Gigante (DG). All Authors oversaw the data acquisition and reviewed the final dataset meticulously checking for inaccuracies and incongruencies. It is also worth highlighting that the shared data are provided raw, without going through any post-processing phase that may alter their soundness. The validity of the data collection is also assured by a set of choices which are detailedly described in the following.

### Location selection

The chosen location is included in the Natura2000 Site designated as SAC IT5210014 “Monti Maggio - Nero (sommità)” in 2014^36^, and, as such, the presence of the target habitat has been previously detected, identified and assessed according to the standard European and national protocols^9,15,37^. Besides wide patches of the target Annex I habitat 6210, the SAC includes four more Annex I habitats, namely 8210, 9210, 9260, 9340 (for a definition of Annex I habitat codes, see^2,15^). Official data on the occurrence and distribution of these habitats in the SAC IT5210014 are part of its Management Plan, published by the Regional Offices of Umbria^38^, and also available in the EU Standard Data Form of the SAC^39^. The habitat maps are part of the Natura 2000 Network Management Plans, adopted by the Umbria Region with^40^ and subsequently approved by the Regional Council. On the field, DG and FB ensured that the chosen plots for data collection are typical examples of habitat 6210.

### Date selection

As described in the Method section, the ideal period to perform the data collection is May. However, being in a natural environment, the precise choice of the best period for sampling mainly depends on the seasonal climatic variations in terms of rain and temperature of the survey area, which influence the vegetative growth. In order to acquire meaningful data, DG and FB tracked the flowering progresses of the target indicator species, starting from mid April, and the dates 10–13 May were selected based on the orchids phenology (e. g. time of full blooming) in the study area.

### On field indicator species classification

The section of the dataset named “Typical and early warning species data” contains pictures and videos of the indicator species. As described in the Method section, the selection of the species which are indicators for habitat 6210 has been performed following articles published on international journals^6,12,14,26,27,30,31^. On the field, plant scientists (DG and FB), who are experts of the habitat 6210 flora, selected and classified the specific instances of the chosen indicator species following the guidelines in^25^.

### Mapping accuracy

The validity of the mapping is directly ensured by the autonomous missions. Indeed, in the mapping phase the robot recorded a digital reconstruction of the surroundings that was then used during the autonomous mission phase. Since the robot did not fail to localize itself in the environment, and it was also able to autonomously walk and perform the monitoring mission, we can infer the point cloud soundness.

### Monitoring mission validity

Monitoring missions have been conducted using methodologies that mimic field relevé performed by botanists to ensure the compliance with national and international standards^9,14,21^. The monitoring algorithm developed by the engineer team establishes that error messages are printed on the terminal when faults occur in the data acquisition process. This fact ensured that no issues were presented during the data acquisition. Additionally, no analysis has been performed on the robot-acquired data. This ensures that no data corruption is present.

### Database inspection

At the end of the data collection, the database has been created. Only valid and complete data have been added to the dataset. Once the dataset was created, both teams carefully revised each entry to check their validity. This means that plant scientists inspected each video and picture of the “Typical and early warning species data” directory to ensure their correct folder placement. Analogously, the robotic engineering team inspected the robot status folders and the point clouds folder to ensure their correct folder placement. In particular, MJP ran test scripts to ensure no corrupted file was present.

## Usage Notes

The presented dataset is highly multidisciplinary and can be employed by researchers working in several different fields. For instance, this dataset can be exploited to achieve the long term goal of the Horizon 2020 Natural Intelligence project (https://www.nih2020.eu/). This is to assist plant scientists in performing habitat monitoring procedures using legged robotic systems. To achieve this objective, two are the main points that need to be tackled: (i) having a robotic platform able to autonomously replicate the survey procedures suggested by national and international standards; and (ii) having algorithms able to (partially) assess the habitat conservation status.

As described in the Background & Summary section, the habitat conservation status can be related to various parameters, among which there is the presence/absence of typical or early warning species^6,8,9,14^. For this reason, to archive point (ii), it is necessary to have algorithms able to classify different plant species. The part of the proposed dataset named “Typical and early warning species” can be used towards this goal. Indeed, it provides labeled pictures and videos which are usually necessary to develop and tune new classifying algorithms. For these reasons, this part of the dataset can be very useful for engineers or computer scientists aiming to design new artificial intelligence based methods for detection and classification of plant species, e.g.^41^. Alternatively, these robot-acquired pictures and videos can be used to validate already existing algorithms that were developed using human-acquired data.

The part of the dataset named “Monitoring mission” can be used to work towards point (i). For instance, LiDAR-acquired point clouds and the robot status can be exploited to advance the research regarding robotic locomotion, navigation, and obstacle avoidance. Additionally, this part of the dataset can be used by botanists to assess the habitat conservation status and to compare them with past or future data from the same plots, or with data from different plots.

The mentioned usages of the dataset are intended as examples. Obviously, many other usages can be found. For instance, instead of using point cloud or robot status data to develop algorithms for habitat monitoring, they can be used to design new methodologies for search&rescue, inspection&maintenance, etc. Indeed, point clouds containing natural environments representations are usually quite useful to validate algorithms tested only in simulation scenarios, decreasing the sim to real gap.

Alternatively, it is possible to exploit the monitoring mission data to build new models. Indeed, these data are georeferenced, so they can be implemented for analysis on remote sensing similarly to^42,43^. The idea is to analyze the images/videos taken by the robot during the autonomous mission to classify the plant species and then integrate this information into species distribution models or validate previously defined models. The position of the plant during the autonomous monitoring mission can be also precisely estimated by merging GPS information with the robot position estimate.

## Data Availability

The MATLAB scripts used to extract and plots data from the ROS bag (.bag) files are provided on the GitHub page of the Research Center E. Piaggio^35^ and are also archived on Zenodo^34^. A description of each script is provided in the README file of the GitHub repository.

## References

[1] Fetting, C. The European Green Deal. *ESDN Report* (European Sustainable Development Network, December, 2020).

[2] European Commission (1992). Council Directive 92/43/ EEC of 21 May 1992 on the conservation of natural habitats and of wild fauna and flora. Official Journal L 206, 22/07/1992. P. 0007–0050. Official Journal of the European Union.

[3] Campagnaro T, Sitzia T, Bridgewater P, Evans D, Ellis EC (2019). Half Earth or whole Earth: what can Natura 2000 teach us?. BioScience.

[4] Evans D (2012). Building the European Union’s Natura 2000 network. Nature Conservation.

[5] Langhout W, Brunner AL (2017). The best idea Europe has ever had? Natura 2000—the largest network of protected areas in the world. The George Wright Forum.

[6] Evans, D. & Arvela, M. Assessment and reporting under article 17 of the habitats directive. explanatory notes & guidelines for the period 2007–2012. *European Commission, Brussels* (European Topic Centre on Biological Diversity, 2011).

[7] Hermoso V, Morán-Ordóñez A, Canessa S, Brotons L (2019). Realising the potential of Natura 2000 to achieve EU conservation goals as 2020 approaches. Scientific Reports.

[8] DG Environment. Reporting under article 17 of the habitats directive: Explanatory notes and guidelines for the period 2013–2018. *Brussels* 1–188 (European Commission, 2017).

[9] Angelini P, Casella L, Grignetti A, Genovesi P (2016). Manuali per il monitoraggio di specie e habitat di interesse comunitario (direttiva 92/43/cee) in italia: habitat. *ISPRA*. Serie Manuali e linee guida.

[10] Wilson JB, Peet RK, Dengler J, Pärtel M (2012). Plant species richness: the world records. Journal of Vegetation Science.

[11] Calaciura, B. & Spinelli, O. Management of Natura 2000 habitats. 6210 Semi-natural dry grasslands and scrubland facies on calcareous substrates (Festuco-Brometalia)(* important orchid sites). *European Commission* 1–38 (2008).

[12] Gigante D (2016). 6210 formazioni erbose secche seminaturali e facies coperte da cespugli su substrato calcareo (festuco-brometalia) semi-natural dry grasslands and scrubland facies on calcareous substrates (festuco-brometalia)(* important orchid sites). Manuali per il monitoraggio di specie e habitat di interesse comunitario (Direttiva 92/43/CEE) in Italia: habitat. ISPRA, Serie Manuali e Linee Guida.

[13] Bonari G (2021). Shedding light on typical species: implications for habitat monitoring. Plant Sociology.

[14] Gigante D (2016). A methodological protocol for Annex I habitats monitoring: the contribution of vegetation science. Plant Sociology.

[15] DG Environment. Interpretation Manual of European Union Habitats. EUR 28. *Nature ENV B.3 Brussels* 144 (European Commission, 2013).

[16] Biondi, E. *et al*. Manuale Italiano di interpretazione degli habitat della Direttiva 92/43/CEE. *Società Botanica Italiana. Ministero dell’Ambiente e della tutela del territorio e del mare, DPN* (2009).

[17] Gijbels P, Adriaens D, Honnay O (2012). An orchid colonization credit in restored calcareous grasslands. Ecoscience.

[18] Koehler M, Elias D, Hiller G, Hoelzel N, Tischew S (2020). Restoration of orchid-rich dry calcareous grasslands by rotational goat pasturing. Tuexenia.

[19] Landi M, Frignani F, Lazzeri C, Angiolini C (2009). Abundance of orchids on calcareous grasslands in relation to community species, environmental, and vegetational conditions. Russian Journal of Ecology.

[20] Dalle Fratte M, Caccianiga M, Ricotta C, Cerabolini BE (2022). Identifying typical and early warning species by the combination of functional-based diagnostic species and dark diversity. Biodiversity and Conservation.

[21] Chytrý M, Otýpková Z (2003). Plot sizes used for phytosociological sampling of european vegetation. Journal of Vegetation Science.

[22] Kettunen, M. *et al*. Assessment of the Natura 2000 co-financing arrangements of the EU financing instrument. *IEEP, Brussels, Belgium* (Institute for European Environmental Policy, 2011).

[23] Sánchez-Fernández D, Abellán P, Aragón P, Varela S, Cabeza M (2018). Matches and mismatches between conservation investments and biodiversity values in the European Union. Conservation Biology.

[24] Hutter M (2017). Anymal-toward legged robots for harsh environments. Advanced Robotics.

[25] Pignatti, S., Guarino, R. & La Rosa, M. Flora d’italia, 2 edizione. *Edagricole di New Business Media, Bologna* (2017–2019).

[26] Gigante, D. *et al*. Manuale diagnostico degli habitat e delle specie nel contesto territoriale umbro. Progetto LIFE13 NAT/IT/000371 SUNLIFE. http://vnr.unipg.it/sunlife/habitat-dettagli.php?id=21 [Online; accessed 15-March-2023] (2015).

[27] Tesei G (2020). Restoration strategies for grasslands colonized by asphodel-dominant communities. Grassland Science.

[28] Bonanomi G, Incerti G, Allegrezza M (2013). Assessing the impact of land abandonment, nitrogen enrichment and fairy-ring fungi on plant diversity of mediterranean grasslands. Biodiversity and Conservation.

[29] Catorci A, Cesaretti S, Gatti R, Ottaviani G (2011). Abiotic and biotic changes due to spread of brachypodium genuense (dc.) roem. & schult. in sub-mediterranean meadows. Community Ecology.

[30] Biondi E (2014). New and validated syntaxa for the checklist of italian vegetation. Plant Biosystems-An International Journal Dealing with all Aspects of Plant Biology.

[31] Allegrezza M, Biondi E, Ballelli S, Tesei G, Ottaviani C (2015). The edge communities of asphodelus macrocarpus subsp. macrocarpus: the different ecological aspects and a new case study in the central apennines. Plant Sociology.

[32] WFO (2022). World flora online. http://www.worldfloraonline.org [Online; accessed 15-March-2023] (2023).

[33] Angelini F, Pollayil MJ, Bonini F, Gigante D, Garabini M (2023). Zenodo.

[34] Pollayil MJ, Angelini F, Garabini M (2023). Zenodo.

[35] Pollayil MJ (2023). Github.

[36] Ministero dell’Ambiente e della Tutela del Territorio e del Mare (MATTM). DECRETO 7 agosto 2014. Designazione di 31 ZSC della regione biogeografica continentale e di 64 ZSC della regione biogeografica mediterranea insistenti nel territorio della Regione Umbria, ai sensi dell’art.3, comma 2, del DPR 8 settembre 1997, n. 357. (14A06579). *GU Serie Generale n.194 del 22-08-2014* (2014).

[37] Biondi E (2012). Diagnosis and syntaxonomic interpretation of Annex I Habitats (Dir. 92/43/EEC) in Italy at the alliance level. Plant Sociology.

[38] Umbria Regional Offices. Management Plan of Special Areas of Conservation IT5210014. https://www.regione.umbria.it/ambiente/siti-di-importanza-comunitaria-sic [Online; accessed 15-March-2023] (2023).

[39] Natura2000. Standard Data Form of SAC IT5210014. https://natura2000.eea.europa.eu/Natura2000/SDF.aspx?site=IT5210014 [Online; accessed 15-March-2023] (2023).

[40] Umbria Region. Delibera Giunta Regionale n. 161 of 08 February 2010. “Piani di Gestione dei siti Natura 2000. Adozione delle proposte di piano e avvio della fase di partecipazione” (2010).

[41] Manh, X. H. *et al*. Towards the computational assessment of the conservation status of a habitat. In *European Conference on Computer Vision*, 751–764 (Springer, 2023).

[42] De Simone W (2021). From remote sensing to species distribution modelling: An integrated workflow to monitor spreading species in key grassland habitats. Remote Sensing.

[43] Ali I, Cawkwell F, Dwyer E, Barrett B, Green S (2016). Satellite remote sensing of grasslands: from observation to management. Journal of Plant Ecology.

